# A Low-Cost Monocular Video-Based Framework for Bilateral Plantar Load Estimation During Treadmill Walking

**DOI:** 10.3390/s26196307

**Published:** 2026-10-06

**Authors:** Yanmei Jiao, Gan Huang, Xuan Huang, Yuan Bian, Bo Wu

**Affiliations:** 1 Department of Computer Science, Graduate School of Bionics, Computer and Media Sciences, Tokyo University of Technology, Hachioji 192-0982, Tokyo, Japan; d212600392@edu.teu.ac.jp (Y.J.); d212600139@edu.teu.ac.jp (G.H.); 2 Graduate School of Human Sciences, Waseda University, Tokorozawa 359-1192, Saitama, Japan; 3 School of Computer Science, Tokyo University of Technology, Hachioji 192-0982, Tokyo, Japan

**Keywords:** gait analysis, machine learning, human motion feature mapping, wearable-sensor-free, plantar load estimation, low-cost

## Abstract

Plantar load is an important biomechanical measure in gait analysis, rehabilitation assessment, and human movement evaluation. However, conventional measurement systems, including instrumented insoles and laboratory-based force measurement devices, rely on dedicated sensing hardware and may limit the accessibility of continuous gait monitoring. This study proposes a low-cost, monocular video-based framework for estimating bilateral plantar loads from pose-derived biomechanical features. As an initial application setting, we focused on treadmill walking, where a stationary camera can capture repeated body motion under consistent recording conditions, allowing the relationship between video-derived biomechanical features and plantar loading to be investigated before extending the framework to more variable environments. Video and reference plantar-load data from 14 healthy adults during treadmill walking at 3 km/h were temporally aligned, yielding 50,464 frame-level observations. Body landmarks estimated using MediaPipe Pose were used to construct posture-related, motion-related, and short-term temporal features. Four machine-learning regressors were evaluated, with fold-specific Top-60 feature selection applied to the morphology-excluded feature configuration. ExtraTrees achieved the highest mean performance under blocked temporal five-fold cross-validation, with $R^{2}$ values of 0.762 ± 0.068 and 0.772 ± 0.073 for the left- and right-foot loads, respectively. Feature-group ablation showed that removing three static morphology variables had only a small effect on blocked-validation performance. Under zero-shot leave-one-subject-out validation, the corresponding mean $R^{2}$ values were −0.252 ± 0.592 and −0.180 ± 0.573, indicating limited generalization to previously unseen participants. Using the initial 30 s of reference-load data for subject-specific supervised calibration improved prediction performance on the matched post-calibration interval. Regularized temporal, scale, and offset calibration increased the mean $R^{2}$ values from −0.233 to 0.358 for the left-foot target and from −0.141 to 0.444 for the right-foot target. These findings demonstrate the potential of monocular video-derived biomechanical features for bilateral plantar-load estimation during controlled treadmill walking. Short supervised calibration improved average prediction performance for unseen participants, enabling subsequent video-based estimation without continuous wearable-sensor measurements.

## 1. Introduction

As global healthcare systems increasingly shift toward home- and community-based care, particularly for aging populations and individuals requiring long-term functional monitoring, accessible and low-cost gait assessment tools are increasingly needed outside conventional clinical settings. Among the various biomechanical parameters used for gait assessment, plantar pressure provides important information regarding foot–ground interaction and lower-limb function during locomotion [1,2].

Plantar pressure distribution describes the spatial and temporal characteristics of the pressure exerted between the plantar surface of the foot and the supporting surface during locomotion [1]. Plantar pressure analysis has been widely applied to gait and posture assessment, footwear evaluation, sports biomechanics, rehabilitation, and injury prevention [1,2]. In clinical practice, plantar pressure measurements can provide objective information for evaluating diabetic foot complications, foot deformities, lower-limb musculoskeletal disorders, and neurological conditions affecting gait and motor function [3,4,5].

Conventional plantar pressure assessment is primarily performed using platform-based pressure measurement systems and instrumented pressure insoles, while force plates are widely used as reference systems for kinetic gait analysis. Platform- and force-plate-based systems can provide accurate measurements under controlled experimental conditions, but their fixed sensing areas and laboratory installation generally restrict data acquisition to relatively short and controlled trials, limiting their suitability for continuous or real-world monitoring [2,6]. Instrumented pressure insoles improve measurement mobility by allowing plantar loading to be recorded across multiple gait cycles and outside fixed laboratory measurement areas [1,7]. Nevertheless, their measurement performance remains dependent on factors such as sensor technology, sensor configuration and placement, calibration, and validation procedures, and considerable heterogeneity exists among currently available in-shoe measurement systems [7]. More broadly, both laboratory-based and wearable pressure measurement approaches remain dependent on dedicated force- or pressure-sensing hardware, presenting practical challenges for scalable and long-term gait monitoring outside controlled environments [6,7].

To reduce dependence on conventional pressure measurement hardware, recent studies have investigated data-driven approaches for estimating plantar pressure and ground reaction forces from wearable inertial sensors or human kinematics. Ren et al. proposed Motion2Press, a cross-modal learning framework that estimates plantar pressure distributions from inertial measurement unit signals [8]. Martínez-Pascual et al. estimated vertical ground reaction forces during gait using lower-limb kinematics and acceleration obtained from wearable inertial sensors [9]. In parallel, vision-based approaches have begun to demonstrate that kinetic information can also be inferred from human motion. Scott et al. developed deep learning models for predicting foot pressure distributions from two- and three-dimensional human pose representations [10], while Feng et al. estimated three-dimensional ground reaction forces and center-of-pressure trajectories from markerless motion-capture-derived kinematics [11].

Recent research on markerless gait analysis further supports the feasibility of extracting human-motion information using camera-based systems. A systematic review and meta-analysis of three-dimensional markerless camera-based gait analysis reported good-to-excellent accuracy, validity, and reliability for multiple spatiotemporal parameters, as well as moderate-to-excellent agreement for hip and knee kinematics, although notable limitations remained for ankle kinematics and some non-sagittal-plane measurements [12]. More specifically, Wang et al. compared a single-camera markerless approach with a marker-based motion-capture system for lower-limb kinematics and spatiotemporal gait parameters, and their findings supported the feasibility of extracting quantitative gait information from single-camera video [13].

These findings support the use of video-derived human pose as a practical input representation for estimating biomechanical variables. However, estimating plantar load from monocular video remains challenging. The mapping from observed body motion to plantar load is complex and may vary across individuals. Participant-specific characteristics and differences in walking patterns may affect plantar-load magnitude and its temporal variation. Regional plantar-pressure distribution is a distinct measurement outcome and is not predicted in the present study. Consequently, a model that performs well on temporally separated data from participants already represented in the training data may not necessarily generalize to previously unseen individuals to the same extent.

In this context, pose-derived biomechanical features provide an interpretable intermediate representation between raw video and plantar-load prediction. Joint configurations, segment motion, and short-term temporal changes characterize complementary aspects of gait dynamics and can be modeled using conventional machine-learning approaches. Previous studies have shown that machine-learning models, including Random Forest and neural-network-based approaches, can estimate ground reaction forces from kinematic inputs, further supporting the general feasibility of mapping motion-derived information to kinetic variables. Compared with fully end-to-end prediction directly from raw images, an explicit feature-based representation also enables direct examination of the contributions of different biomechanical feature groups to model prediction.

Accordingly, this study proposes a monocular video-based machine-learning framework for estimating bilateral body-mass-scaled plantar loads from pose-derived biomechanical features. Synchronized video and plantar-loading data were collected from 14 healthy participants during controlled treadmill walking at 3 km/h. Three-dimensional body keypoints were extracted using MediaPipe Pose and further transformed into biomechanical features representing posture, motion, and short-term temporal dynamics. An ExtraTrees regression model with fold-specific feature selection was then used to estimate body-mass-scaled left- and right-foot plantar loads.

The proposed framework was evaluated from several complementary perspectives to assess both predictive performance and generalization capability. First, blocked temporal five-fold cross-validation with a 2 s temporal gap was used to evaluate prediction performance on temporally separated walking segments within the studied participant population, while leave-one-subject-out validation was used to assess generalization to previously unseen participants. Second, feature-group ablation was performed to investigate the relative contributions of posture, motion, and morphological information to plantar-load estimation. Finally, because inter-individual differences may affect the pose-to-load relationship, a short subject-specific supervised calibration procedure was further investigated to determine whether low-dimensional scale, offset, and temporal adjustments could improve personalized prediction for new participants.

The main contributions of this study are summarized as follows:

1. A monocular video-based framework is proposed for estimating bilateral body-mass-scaled plantar loads from pose-derived biomechanical features, providing a low-cost approach to camera-based gait assessment.
2. An interpretable biomechanical feature representation incorporating posture, motion, and short-term temporal information is constructed from body-pose trajectories estimated from monocular video, and fold-specific feature selection is combined with ExtraTrees regression to estimate bilateral plantar load.
3. The proposed method is evaluated under both temporally separated and subject-independent validation settings, enabling separate assessment of predictive performance within the studied participant population and generalization to previously unseen individuals.
4. A low-dimensional subject-specific supervised calibration strategy based on scale, offset, and temporal adjustment is investigated to compensate for inter-individual differences in unseen participants and improve personalized plantar-load prediction while preserving the interpretability of the calibration parameters.

## 2. Related Works

### 2.1. Plantar Pressure Measurement Methods

Plantar pressure measurement provides quantitative information on foot–ground interaction and is widely used in gait assessment, rehabilitation, sports biomechanics, and clinical monitoring [1,7]. Conventional approaches mainly include platform-based systems and in-shoe pressure systems.

Platform-based systems provide detailed plantar pressure measurements under controlled laboratory conditions, but their fixed sensing area limits data collection to a small number of steps and reduces their suitability for continuous gait monitoring [14]. In contrast, in-shoe systems integrate pressure sensors into footwear and allow plantar loading to be recorded over multiple gait cycles during more natural activities [7].

Despite improvements in sensor technology and portability, existing systems still depend on specialized pressure-sensing hardware. Their practical use can also be affected by sensor configuration, calibration procedures, device design, and cross-system measurement differences [15,16]. These limitations increase system cost and hardware dependence, particularly when repeated or large-scale gait assessment is required.

Therefore, there is growing motivation to develop low-cost and wearable-sensor-free alternatives that can estimate plantar loading without requiring pressure-sensing devices during inference. In this context, extracting human motion information from ordinary video and using these features to estimate bilateral plantar loads through machine learning represents a promising direction for more accessible gait analysis.

### 2.2. Wearable Sensor-Based Estimation of Plantar Pressure and Ground Reaction Forces

Wearable sensing has become an important alternative for estimating kinetic gait variables outside conventional laboratory systems. Inertial measurement units (IMUs), in particular, can capture segment acceleration and orientation and have been combined with machine-learning models to estimate ground reaction forces from body-motion information.

Martínez-Pascual et al. estimated vertical ground reaction forces during walking using lower-limb kinematics and acceleration measured by wearable inertial sensors, demonstrating that external loading can be reconstructed from wearable motion data [9]. Similarly, Carter et al. showed that consumer-priced wearable sensors combined with deep learning could predict continuous ground reaction forces across different treadmill running conditions [17].

More recently, wearable sensing has been extended from GRF estimation to plantar-pressure-related variables. Ren et al. proposed Motion2Press, which uses IMU signals and cross-modal learning to infer plantar pressure distribution, GRF, and center of pressure (COP) [8]. A systematic review of neural-network-based plantar pressure analysis also identified wearable sensing and IMUs as increasingly important sources of information for plantar pressure estimation and gait analysis [16].

Although these approaches reduce dependence on fixed laboratory equipment, they still require sensors to be worn on the body and may involve sensor placement, calibration, synchronization, and user-compliance requirements. These limitations motivate the development of wearable-sensor-free approaches that estimate plantar loading from externally observed human motion.

### 2.3. Markerless Pose Estimation for Gait Analysis

Markerless pose estimation has increasingly been applied to gait analysis as a low-cost alternative to conventional marker-based motion-capture systems. Recent systematic evidence indicates that computer-vision-based gait analysis can provide clinically meaningful kinematic and spatiotemporal information, although performance varies across algorithms, joints, and experimental conditions [17].

OpenPose-based systems have been validated for treadmill walking and running, showing good agreement with reference motion-capture systems for sagittal-plane lower-limb joint motion, while larger errors have been reported for frontal-plane measurements [18]. Similarly, low-cost multi-camera systems combining OpenPose with ordinary RGB webcams have demonstrated the feasibility of reconstructing three-dimensional gait kinematics without reflective markers [19].

More recent studies have extended markerless gait analysis to simplified camera setups. Automated gait-analysis frameworks based on pose estimation have been developed for extracting gait parameters from video [20], while smartphone-based systems have shown that running gait characteristics can be assessed using a single consumer camera [21].

### 2.4. Machine Learning for Biomechanical Prediction

Machine-learning methods have increasingly been applied to biomechanical prediction tasks because they can model complex relationships between human motion and kinetic variables that are difficult to represent using simple analytical equations. Recent studies have demonstrated that machine learning can estimate ground reaction forces, joint moments, and center-of-pressure variables from kinematic, inertial, and markerless motion data [11,22].

Young et al. demonstrated the feasibility of markerless running gait assessment using a single smartphone camera within an Internet-of-Things-enabled framework [23]. Bogaert et al. used machine learning to predict key vertical ground reaction force characteristics during running from three-dimensional sacral acceleration, showing that data-driven models can recover clinically relevant loading variables from motion-related inputs [24]. More recently, Feng et al. applied multilayer perceptron and convolutional neural network models to markerless motion-capture trajectories and successfully estimated three-dimensional GRF and COP during walking [11].

Broader evidence also supports the increasing role of AI-based models in biomechanical prediction. A recent systematic review of lower-limb joint-moment prediction reported that both deep-learning and conventional machine-learning approaches can achieve useful predictive performance, although results depend strongly on the input modality, model architecture, and validation strategy [25]. A broader narrative review further highlighted the expanding application of artificial intelligence in biomechanical assessment, diagnosis, and physical rehabilitation [26]. In addition, a recent scoping review of machine-learning approaches for predicting lower-extremity joint contact loads identified persistent challenges related to limited dataset sizes, model generalization, and validation methodology [27]. Complementary to these data-driven approaches, biomechanically informed methods have also been investigated for kinetic prediction. Wagner et al. examined the virtual pivot point concept for improving ground reaction force prediction, illustrating the potential value of incorporating biomechanical principles into predictive modeling [28].

These findings support the use of machine learning to estimate kinetic gait variables from human-motion information. However, relatively few studies have systematically compared conventional machine-learning models using interpretable pose-derived biomechanical features. Moreover, model performance can depend substantially on the validation strategy, and results obtained from participants represented during training may not reflect performance on previously unseen individuals. These considerations motivate the model comparison, feature-group analysis, subject-independent validation, and subject-specific calibration investigated in the present study.

## 3. Methodology

This study proposes a Monocular Pose-to-Load Framework (MPLF) for estimating bilateral plantar load from biomechanical features derived from monocular walking videos. The framework integrates video-based body-pose estimation, pose-trajectory preprocessing, biomechanical and temporal feature construction, machine-learning-based prediction, and optional short-term subject-specific calibration.

The present study focuses on normal treadmill walking and predicts two target variables: left plantar load ($L_{\mathrm{Load}}$) and right plantar load ($R_{\mathrm{Load}}$). These variables represent the overall loads measured separately beneath the left and right feet.

To investigate the performance and transferability of the proposed framework, two complementary validation strategies were adopted. Blocked temporal five-fold cross-validation was used to evaluate predictions on temporally separated walking observations from participants represented during model development. Leave-one-subject-out (LOSO) validation was used to evaluate predictions for participants entirely excluded from model training. Short-term supervised calibration was subsequently investigated as an optional strategy for adapting the predictions of a trained population model to a previously unseen participant.

### 3.1. Framework Overview

**Figure 1:**
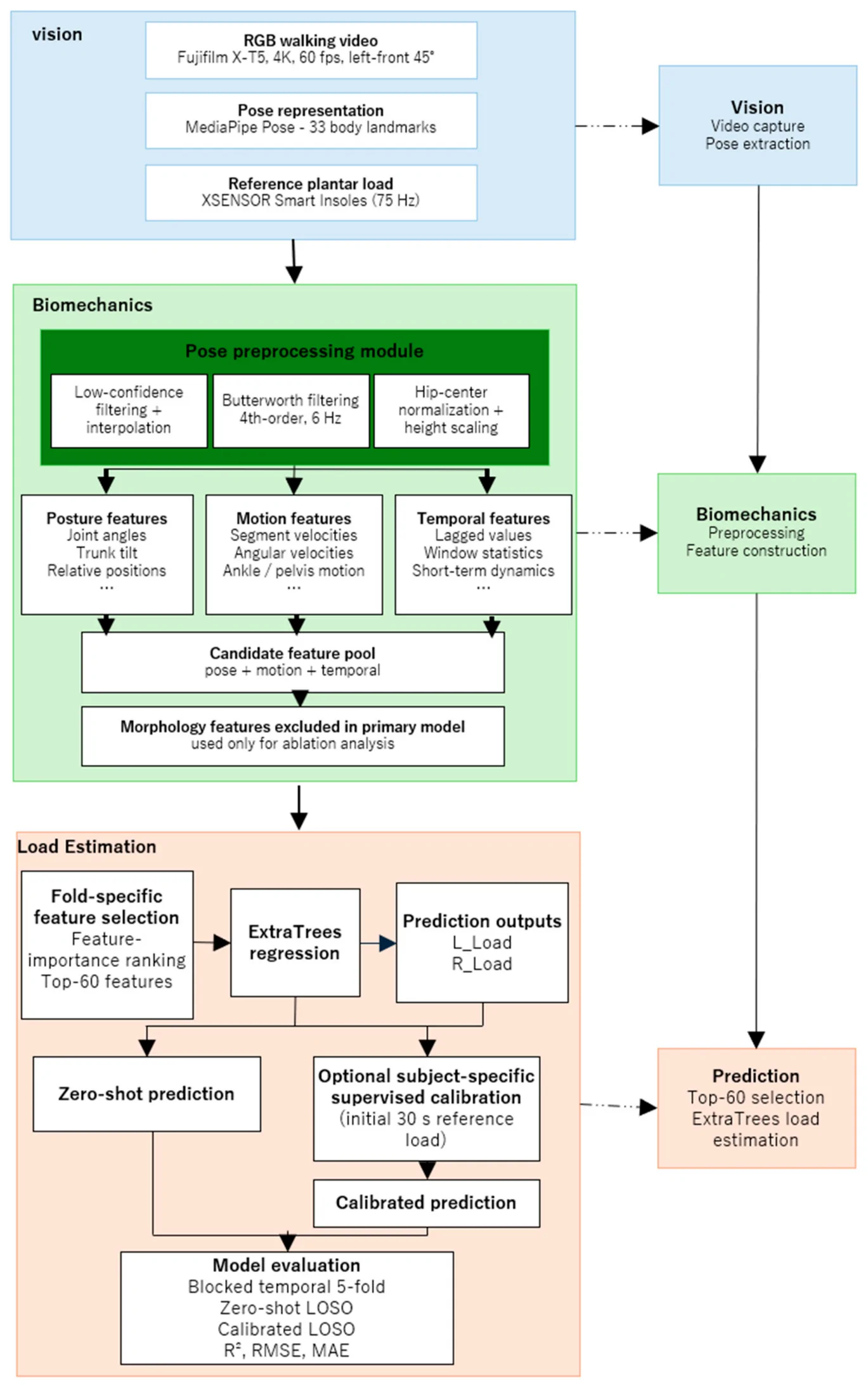
Overview of the proposed Monocular Pose-to-Load Framework (MPLF), including video-based pose estimation, trajectory preprocessing, biomechanical and temporal feature construction, fold-specific feature selection, ExtraTrees regression, model validation, and optional short-term subject-specific calibration. Dashed arrows indicate the correspondence between the detailed processing modules and the three summarized framework stages shown on the right. Ellipses (“...”) indicate that only representative features are listed and that additional features are included in the corresponding feature group.

As illustrated in Figure 1, the proposed Monocular Pose-to-Load Framework (MPLF) comprises three main modules: vision-based data acquisition and pose extraction, biomechanical feature construction, and machine-learning-based plantar-load estimation. The framework uses human-motion information derived from monocular walking videos to estimate bilateral plantar load and incorporates an optional subject-specific calibration procedure for personalized prediction.

In the vision module, walking videos are acquired using a single RGB camera, and MediaPipe Pose is employed to extract body landmarks from the recorded frames. Reference plantar-load measurements are collected using XSENSORSmartInsoles (XSENSOR Technology Corporation, Calgary, AB, Canada) and temporally aligned with the video-derived observations. These measurements serve as ground-truth data for model development and evaluation and are additionally used during the optional supervised calibration procedure.

In the biomechanics module, the estimated pose trajectories undergo preprocessing to handle unreliable observations, reduce measurement noise, and normalize body coordinates. Biomechanical features are then constructed to characterize body posture, segment motion, and short-term temporal dynamics. Static morphological features are excluded from the primary prediction configuration and retained for feature-group ablation analysis.

In the load-estimation module, feature selection is performed independently within each training fold, and the 60 highest-ranked features are used to train separate ExtraTrees regression models for the left and right plantar-load targets ($L_{\mathrm{Load}}$ and $R_{\mathrm{Load}}$). For previously unseen participants, the trained population model can be applied directly to generate zero-shot predictions or optionally adapted through a 30 s supervised subject-specific calibration procedure. The latter uses an initial period of reference plantar-load measurements to estimate individual adjustment parameters, which are subsequently applied to the model predictions.

Model performance is evaluated using blocked temporal five-fold cross-validation and leave-one-subject-out (LOSO) validation. The blocked temporal protocol assesses prediction on temporally separated observations from participants represented during training, whereas LOSO validation assesses zero-shot and calibrated prediction for previously unseen participants. The two LOSO prediction settings are compared over the same post-calibration evaluation interval.

### 3.2. Experimental Setup and Protocol

**Figure 2:**
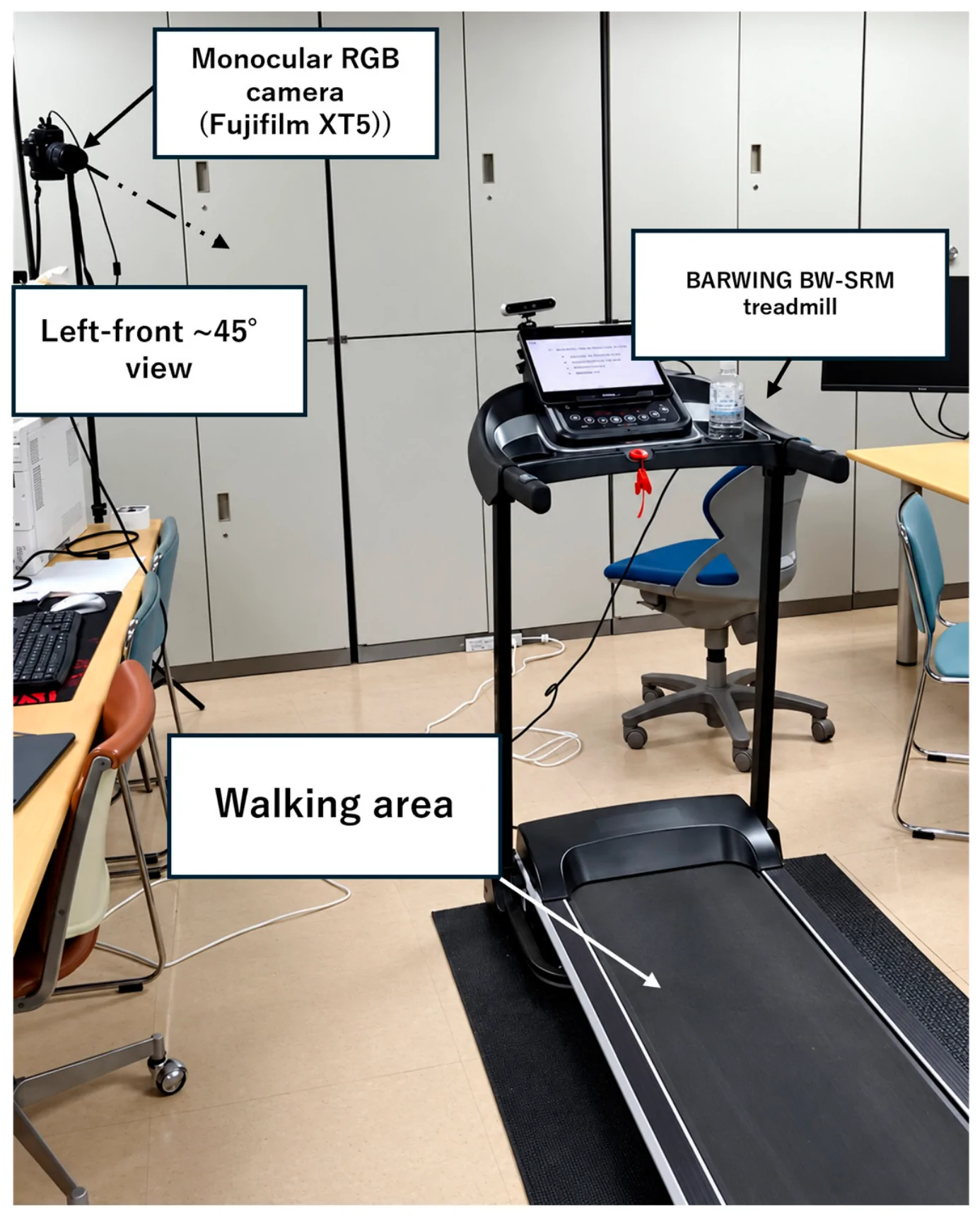
Experimental environment and recording setup. The dashed arrow indicates the approximate left-front 45° viewing direction of the monocular RGB camera.

The experiments were conducted in a controlled university laboratory, as shown in Figure 2, using a BARWING BW-SRM treadmill (BW-SRM, All Market Japan Co., Ltd., Tatebayashi, Japan). The treadmill incline was fixed at 0° throughout data collection. To reduce footwear-related variability, all participants wore standardized flat-soled shoes with hard rubber soles.

Reference plantar-load measurements were acquired using XSENSOR Smart Insoles (XSENSOR Technology Corporation, Calgary, AB, Canada) at a sampling frequency of 75 Hz.

Human-motion videos were recorded using a Fujifilm X-T5 camera (FUJIFILM Corporation, Tokyo, Japan) in a monocular RGB configuration at approximately 60 frames per second. As shown in Figure 2, the camera was mounted on a fixed tripod and positioned on the participant’s left-front side, approximately 1.5–2.0 m from the treadmill and approximately 1.65 m above the ground, forming an observation angle of approximately 45°. The recording position was adjusted to ensure full-body visibility during treadmill walking. Figure 2 also illustrates the overall laboratory environment, including the treadmill, the camera setup, and the surrounding workspace.

A total of 14 healthy adult participants were included in the present analysis. Although the broader experimental protocol included additional walking conditions and upper-body activity tasks, the present study focused exclusively on normal treadmill walking at 3 km/h. Data collected under the other conditions were not included in the model development and evaluation reported here.

Data acquisition began only after the treadmill reached the target speed and the participant established a stable walking pattern. Video and plantar-load recordings were acquired as separate data streams and were subsequently aligned during data preparation.

The final analysis dataset comprised 50,464 frame-level observations from the 14 participants under the normal 3 km/h treadmill-walking condition.

### 3.3. Data Synchronization and Preprocessing

Body-pose information was extracted from the monocular walking videos using MediaPipe Pose (version 0.10.21), which estimates 33 body landmarks for each frame. The resulting landmark trajectories were used as the basis for subsequent biomechanical feature construction. Because the coordinates were inferred from monocular video, they represent video-estimated body-pose information rather than directly measured three-dimensional marker positions.

The extracted pose trajectories were preprocessed to reduce noise, handle unreliable observations, and limit the influence of global coordinate position and participant body size. Landmark coordinates were smoothed using a fourth-order Butterworth low-pass filter with a cutoff frequency of 6 Hz. Forward–backward filtering was performed using filtfilt with a pose sampling-frequency setting of 59.94 Hz, providing zero-phase filtering within each valid trajectory segment. Missing observations and changes in trajectory-segment identifiers were treated as segment boundaries to prevent filtering across discontinuities, and segments containing fewer than 36 frames were left unfiltered.

Following filtering, pose coordinates were translated relative to the midpoint of the left and right hip landmarks. The hip-center position at time t was defined as

(1) $$p_{hip}\left(t\right)=\frac{p_{LHip}\left(t\right)+p_{RHip}\left(t\right)}{2}$$

where $p_{LHip}\left(t\right)$ and $p_{RHip}\left(t\right)$ denote the estimated positions of the left and right hip landmarks, respectively. Each landmark coordinate was then normalized according to

(2) $${\tilde{p}}_{j}\left(t\right)=\frac{p_{j}\left(t\right)-p_{hip}\left(t\right)}{h_{s}}$$

where $p_{j}\left(t\right)$ is the estimated position of landmark j, and $h_{s}$ denotes the recorded height of participant s. This transformation expresses landmark positions relative to the hip center and scales the coordinates by participant height. Observations identified as invalid by the preprocessing quality flags were excluded from subsequent feature construction.

Video-derived observations and plantar-load measurements were acquired at different sampling frequencies and were aligned during data preparation to establish temporal correspondence between pose-derived inputs and reference plantar-load measurements. The resulting paired observations were used for feature construction, model training, and evaluation. The temporal adjustment investigated later in the subject-specific calibration experiments was applied to the model predictions and was treated as an empirical adjustment of the correspondence between predicted and reference waveforms, rather than as a direct measurement of the original acquisition-system synchronization error.

The XSENSOR system provided estimated load measurements for the left and right insoles in pounds-force (lbf). To construct the prediction targets, each foot-load value was divided by the corresponding participant’s body mass in kilograms:

(3) $$L_{Load}\left(t\right)=\frac{F_{L}\left(t\right)}{m_{s}},\;R_{Load}\left(t\right)=\frac{F_{R}\left(t\right)}{m_{s}}$$

where $F_{L}\left(t\right)$ and $F_{R}\left(t\right)$ denote the estimated left and right foot loads in lbf, and $m_{s}$ is the body mass of the participant ***s*** in kg. Accordingly, $L_{Load}$ and $R_{Load}$ are referred to as body-mass-scaled plantar-load variables, with numerical units of lbf/kg, and should not be interpreted as dimensionless fractions of body weight. These two variables were used as the prediction targets throughout the present study.

### 3.4. Pose-Derived Biomechanical Feature Construction

The preprocessed and body-normalized pose trajectories described in Section 3.3 were used to construct biomechanical features representing instantaneous body configuration, segment motion, and short-term movement history. Feature construction was performed independently within each participant and trial so that motion information was not combined across different walking sequences.

First, a set of 30 core biomechanical variables was derived from the normalized pose coordinates. These variables consisted of eight posture-related features, nineteen motion-related features, and three morphology-related features. The variables included in each category are summarized in Table 1. The feature groups used in the subsequent experiments were explicitly defined in the analysis implementation.

Posture-related features were calculated from the spatial relationships among the estimated body landmarks. Bilateral knee angles were derived from the hip–knee–ankle configurations, whereas bilateral hip angles were derived from the shoulder–hip–knee configurations. Trunk orientation was represented by the relative orientation of the shoulder center and hip center. In addition, the relative vertical positions of the left and right ankles and the distance between the two ankle landmarks were calculated to represent lower-limb configuration.

Motion-related features were derived from temporal changes in the normalized pose trajectories. Translational motion was characterized using the pelvis, knee, and ankle trajectories. For the pelvis, left and right knees, and left and right ankles, velocity-related variables were calculated to represent overall translational motion and vertical movement. Vertical acceleration variables were further derived from changes in the corresponding vertical velocities. Angular velocities of the bilateral knee and hip angles were also calculated to characterize temporal changes in lower-limb joint configuration.

Three relatively stable geometric variables—trunk length, hip width, and shoulder width—were additionally calculated to represent body morphology. These variables were retained for the feature-group ablation experiments but were excluded from the primary prediction configuration to reduce dependence on static participant-specific body dimensions.

To incorporate recent movement history, selected posture and motion variables were further expanded using short-term temporal features. Four temporal scales were considered: 0.1, 0.2, 0.5, and 0.8 s. Three types of temporal representation were used: lagged values, rolling means, and rolling standard deviations. The implementation explicitly specifies the variables used for lag and rolling feature construction.

For a biomechanical variable x, the lagged feature at temporal offset Δt was defined as

(4) $$x_{\mathrm{lag},\Delta t}\left(t\right)=x\left(t-\Delta t\right)$$

where $x\left(t\right)$ denotes the current value and $x\left(t-\Delta t\right)$ represents the value observed Δt seconds earlier.

Lagged features were generated for 14 variables: bilateral knee angles, bilateral hip angles, trunk tilt angle, bilateral relative ankle heights, ankle distance, pelvis velocity magnitude, bilateral ankle velocity magnitudes, bilateral ankle vertical velocities, and pelvis vertical velocity.

To characterize the local level and variability of recent movement, backward-looking rolling statistics were additionally calculated. For a temporal window containing $N_{\Delta t}$ observations, the rolling mean was defined as

(5) $$\mu_{\Delta t}\left(t\right)=\frac{1}{N_{\Delta t}}\sum_{k=0}^{N_{\Delta t}-1}x\left(t-k\right)$$

And the rolling standard deviation was defined as

(6) $$\sigma_{\Delta t}\left(t\right)=\sqrt{\frac{1}{N_{\Delta t}-1}\sum_{k=0}^{N_{\Delta t}-1}{\left[x\left(t-k\right)-\mu_{\Delta t}\left(t\right)\right]}^{2}}$$

Rolling mean and standard-deviation features were generated for seven variables: bilateral knee angles, bilateral relative ankle heights, pelvis velocity magnitude, and bilateral ankle velocity magnitudes.

Temporal features were constructed separately within each participant–trial sequence so that history information did not cross participant or trial boundaries. For the 0.8 s temporal representation, the corresponding number of samples was determined from the median sampling interval of each individual sequence, and the resulting lagged and rolling features were generated using backward-looking observations.

At each temporal scale, 14 lagged features, seven rolling-mean features, and seven rolling-standard-deviation features were generated, giving 28 temporal features per scale. Across the four temporal scales, the temporal expansion produced 112 features. Together with the 30 core biomechanical variables, the complete candidate feature pool therefore contained 142 features.

For the primary MPLF configuration, the three static morphology variables (trunk_length, hip_width, and shoulder_width) were excluded. Because these variables were not used as sources for temporal feature construction, their removal resulted in a 139-feature primary candidate pool consisting of posture-, motion-, and short-term temporal information.

### 3.5. Model Selection and ExtraTrees Regression

To determine an appropriate regression model for plantar-load estimation, four tree-based machine-learning approaches—LightGBM, XGBoost, CatBoost, and ExtraTrees—were compared under the same blocked temporal cross-validation framework using identical data partitions and evaluation metrics. The comparison was designed to examine the ability of different tree-based learning strategies to model the nonlinear relationship between pose-derived biomechanical features and plantar-load measurements. Based on this comparison, ExtraTrees was selected as the principal regression model for the subsequent analyses.

Before regression modeling, feature selection was performed independently within each training fold. The primary feature pool contained 139 candidate features. For each validation fold and each plantar-load target, a LightGBM regressor was fitted exclusively to the corresponding training data and used to rank the candidate predictors according to feature importance. The 60 highest-ranked features were then retained for subsequent regression modeling.

The LightGBM model used for feature ranking was configured with n_estimators = 60, learning_rate = 0.07, num_leaves = 25, subsample = 0.90, and colsample_bytree = 0.90. These settings correspond to the fold-specific feature-selection implementation used in the principal blocked temporal and zero-shot LOSO analyses.

Feature ranking was repeated independently for every validation fold and separately for $L_{\mathrm{Load}}$ and $R_{\mathrm{Load}}$. Thus, the selected Top-60 feature subset was not globally fixed and could vary across folds and between the two prediction targets.

Following feature selection, separate ExtraTrees regressors were trained for the left- and right-foot plantar-load targets. ExtraTrees constructs an ensemble of randomized decision trees and was used to model nonlinear relationships among the selected biomechanical and temporal predictors.

The principal ExtraTrees hyperparameters were set as shown in Table 2.

### 3.6. Model Validation and Feature-Group Ablation

Two complementary validation strategies were used to evaluate the proposed model under different generalization settings: blocked temporal five-fold cross-validation and zero-shot leave-one-subject-out (LOSO) validation.

For blocked temporal five-fold cross-validation, the observations within each participant’s walking trial were divided into five contiguous temporal regions. In each fold, one region was reserved for validation, while the remaining eligible observations were used for model training. A 2 s exclusion gap was introduced on both sides of the held-out temporal region so that observations immediately adjacent to the validation data were not included in the training set. Feature selection and model fitting were repeated independently within each fold. Because data from the same participants were represented in both the training and validation sets, this protocol evaluated prediction on temporally separated observations from participants already represented during model development rather than subject-independent generalization.

Zero-shot LOSO validation was used to evaluate prediction for previously unseen participants. In each LOSO fold, data from 13 participants were used for feature selection and model training, while the remaining participant was completely held out for evaluation. No data from the held-out participant were used during feature selection or ExtraTrees model fitting, and no reference plantar-load measurements from that participant were used to estimate participant-specific adjustment parameters under the zero-shot condition. The procedure was repeated until each of the 14 participants had served once as the held-out participant.

Thus, the two validation protocols addressed different prediction settings: blocked temporal cross-validation assessed performance on new temporal segments from represented participants, whereas zero-shot LOSO assessed direct transfer to participants who were not included in model development.

In addition, feature-group ablation experiments were conducted to examine the predictive contribution of different categories of pose-derived information. Five feature configurations were evaluated: all features, features excluding morphology, posture-related features only, motion-related features only, and morphology-related features only. Feature selection and ExtraTrees training were repeated independently for each configuration.

### 3.7. Subject-Specific Supervised Calibration

Zero-shot LOSO validation was complemented by a 30 s subject-specific supervised calibration procedure.

For each LOSO fold, the population model was first trained using data from the other 13 participants. The initial 30 s of walking data from the held-out participant were then used as the calibration interval. During this interval, the population-model predictions and the corresponding reference plantar-load measurements were used to estimate participant-specific calibration parameters. The ExtraTrees model itself was not retrained. Calibration was performed separately for the left- and right-foot plantar-load predictions.

The first calibration strategy applied a participant-specific scale and offset:

(7) $${\hat{y}}_{\mathrm{cal}}\left(t\right)=a{\hat{y}}_{\mathrm{global}}\left(t\right)+b$$

where ${\hat{y}}_{\mathrm{global}}\left(t\right)$ denotes the original population-model prediction, a is the scale parameter, and b is the additive offset. Regularization was applied during parameter estimation.

The second strategy additionally introduced a temporal adjustment parameter τ:

(8) $${\hat{y}}_{\mathrm{cal}}\left(t\right)=a{\hat{y}}_{\mathrm{global}}\left(t-\tau \right)+b$$

where τ adjusts the temporal alignment between the predicted and reference plantar-load waveforms. Candidate temporal shifts within approximately ±300 ms were evaluated using the calibration interval, together with regularized scale and offset estimation.

Zero-shot and calibrated predictions were evaluated over the same post-calibration time interval. The initial 30 s calibration period was excluded from evaluation, and boundary regions corresponding to the maximum permitted temporal adjustment were removed so that all methods were assessed using identical evaluation timestamps.

The temporal calibration procedure was evaluated offline. After the initial calibration interval, subsequent calibrated predictions were generated from the video-derived model outputs without continuous plantar-load measurement.

### 3.8. Evaluation Metrics

Prediction performance was evaluated separately for the left- and right-foot plantar-load targets using the coefficient of determination ($R^{2}$), root mean squared error (RMSE), and mean absolute error (MAE).

The coefficient of determination was calculated as

(9) $$R^{2}=1-\frac{\sum_{i=1}^{n}{\left(y_{i}-{\hat{y}}_{i}\right)}^{2}}{\sum_{i=1}^{n}{\left(y_{i}-\overset{-}{y}\right)}^{2}}$$

where $y_{i}$ and ${\hat{y}}_{i}$ denote the reference and predicted plantar-load values, respectively, $\overset{-}{y}$ is the mean reference value of the evaluated observations, and n is the number of evaluated samples.

RMSE was calculated as

(10) $$\mathrm{RMSE}=\sqrt{\frac{1}{n}\sum_{i=1}^{n}{\left(y_{i}-{\hat{y}}_{i}\right)}^{2}\;\;\;}$$

And MAE as

(11) $$\mathrm{MAE}=\frac{1}{n}\sum_{i=1}^{n}|y_{i}-{\hat{y}}_{i}|$$

RMSE and MAE were reported in the same units as the processed plantar-load targets.

For blocked temporal five-fold cross-validation, the metrics were calculated for each validation fold and summarized using the mean and standard deviation. For LOSO validation, the metrics were calculated separately for each held-out participant and then summarized across the 14 participants using the mean and standard deviation. For subject-specific calibration, zero-shot and calibrated predictions were evaluated over the same post-calibration interval.

## 4. Dataset and Results

### 4.1. Participants and Ethical Approval

The original treadmill-walking experiment involved participants with different health conditions. For the present analysis, data from 14 healthy adult participants were selected. Their ages ranged from 22 to 34 years, their heights ranged from 165.0 to 187.5 cm, and their body weights ranged from 55 to 102 kg.

Although the original experimental protocol included multiple walking conditions, the present analysis was restricted to normal treadmill walking at a speed of 3 km/h. Data from participants not included in the healthy-participant subset and data collected under other experimental conditions were excluded from the present analysis.

Before the experiments, participants received an explanation of the study objectives, experimental procedures, and potential risks, and provided written informed consent. The study protocol was reviewed and approved by the Ethics Review Committee for Research Involving Human Subjects at Waseda University, with approval number 2025-656.

### 4.2. Data Processing

Following data selection and preprocessing, the final dataset comprised 50,464 frame-level observations from 14 healthy adult participants during normal treadmill walking at 3 km/h.

A total of 30 core biomechanical variables were extracted from the video-estimated pose trajectories. Short-term temporal features were subsequently constructed using lagged observations and backward-looking rolling statistics. After excluding the three static morphology variables, the primary model configuration contained 139 candidate pose-derived features.

The prediction targets were the body-mass-scaled left- and right-foot plantar loads, denoted as $L_{\mathrm{Load}}$ and $R_{\mathrm{Load}}$, respectively.

### 4.3. Model Comparison

Four tree-based regression models—LightGBM(version 4.7.0), XGBoost (version 3.2.0), CatBoost (version 1.2.10), and ExtraTrees (scikit-learn version 1.9.0)—were evaluated using blocked temporal five-fold cross-validation with a 2 s exclusion gap. All models were evaluated using the morphology-excluded feature configuration, with the same training and validation samples and fold-specific Top-60 selected features. The mean prediction performance across the five folds is summarized in Table 3.

ExtraTrees achieved the highest mean $R^{2}$ and the lowest RMSE and MAE for both plantar-load targets. For $L_{\mathrm{Load}}$, ExtraTrees achieved an $R^{2}$ of 0.762±0.068, an RMSE of 0.165±0.023, and an MAE of 0.117±0.016. For $R_{\mathrm{Load}}$, the corresponding values were 0.772±0.073, 0.159±0.024, and 0.110±0.015, respectively. The blocked temporal validation results showed similar prediction performance for the left- and right-foot plantar-load targets.

LightGBM, XGBoost, and CatBoost achieved lower mean $R^{2}$ values than ExtraTrees for both targets, as detailed in Table 3. ExtraTrees was selected as the principal regression model for the subsequent zero-shot LOSO validation, feature-group ablation, and subject-specific calibration analyses.

### 4.4. Zero-Shot LOSO Performance

Zero-shot leave-one-subject-out (LOSO) validation was conducted to evaluate the performance of the primary ExtraTrees model on previously unseen participants. In each fold, the model was trained using data from 13 participants and evaluated on the remaining participant without subject-specific calibration. The results across all 14 held-out participants are summarized in Table 4.

The mean $R^{2}$ values were −0.252 ± 0.592 for $L_{\mathrm{Load}}$ and −0.180 ± 0.573 for $R_{\mathrm{Load}}$. The corresponding RMSE values were 0.364 and 0.350, while the MAE values were 0.331 and 0.312, respectively. Both targets showed lower mean $R^{2}$ values under zero-shot LOSO validation than under blocked temporal cross-validation.

**Figure 3:**
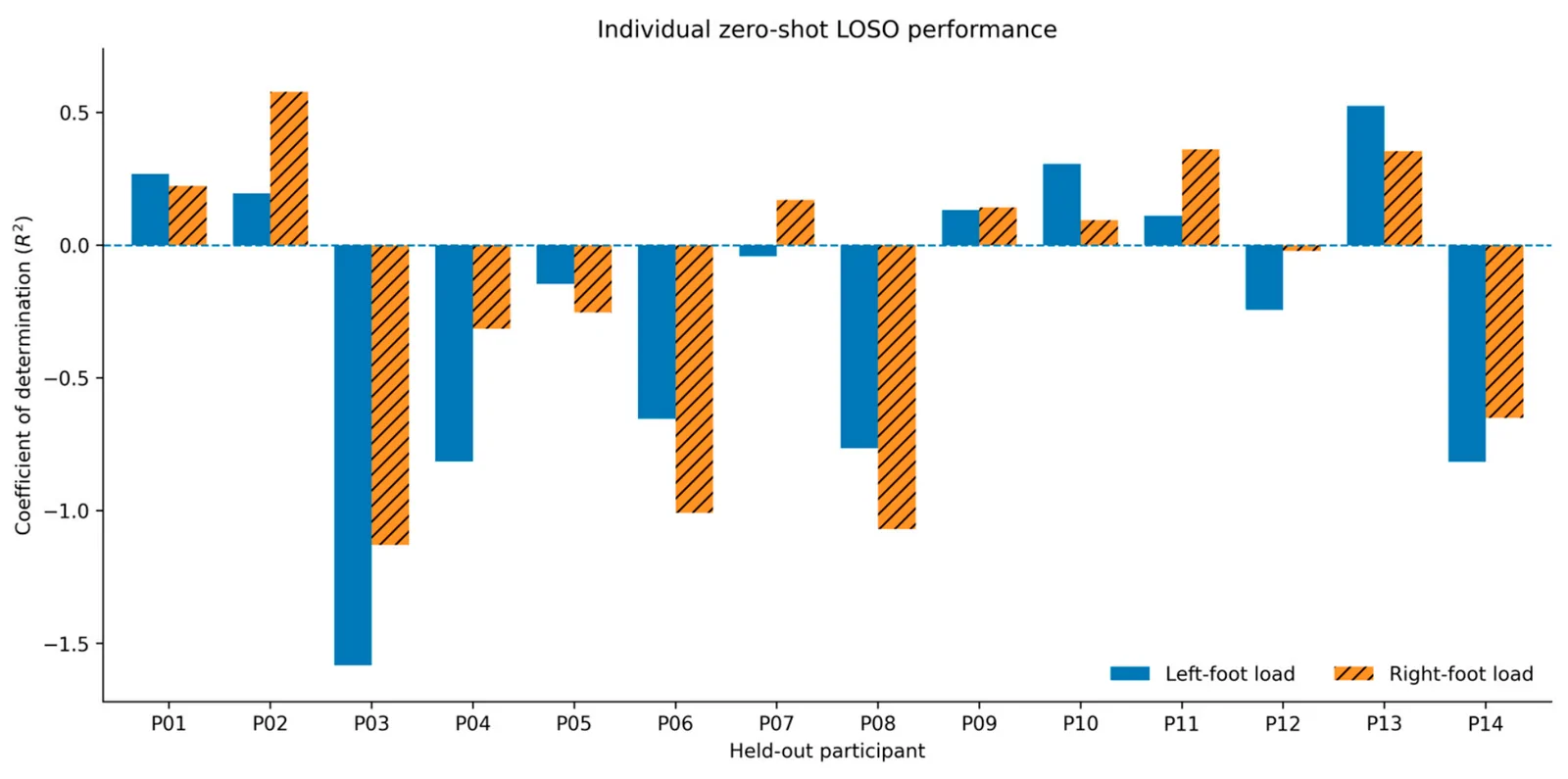
Individual-level coefficient of determination ($R^{2}$) for left- and right-foot plantar-load prediction under zero-shot LOSO validation. Each participant was held out from model training and feature selection. The dashed horizontal line indicates $R^{2}=0$.

Figure 3 presents the individual $R^{2}$ values for the left- and right-foot plantar-load targets. Prediction performance varied across participants. Positive $R^{2}$ values were obtained for 6 of the 14 participants for $L_{\mathrm{Load}}$ and 7 of the 14 participants for $R_{\mathrm{Load}}$. The remaining participants had $R^{2}$ values below zero for the corresponding targets.

### 4.5. Feature-Group Ablation Results

Feature-group ablation was performed using five configurations: all features, features without morphology, posture-related features only, motion-related features only, and morphology-related features only. Each configuration was evaluated under both blocked temporal cross-validation and zero-shot LOSO validation, as summarized in Table 5.

Under blocked temporal validation, similar performance was obtained using all features, the morphology-excluded configuration, and the posture-only configuration. The all-feature configuration achieved mean $R^{2}$ values of approximately 0.768 and 0.777 for the left and right targets, respectively. After excluding the three static morphology variables, the corresponding values were approximately 0.762 and 0.772. The posture-only configuration achieved approximately 0.773 and 0.777.

Lower blocked-validation performance was obtained using motion-related features alone, with mean $R^{2}$ values of approximately 0.577 for $L_{\mathrm{Load}}$ and 0.621 for $R_{\mathrm{Load}}$. The morphology-only configuration produced substantially lower values of approximately 0.097 and 0.094.

Under zero-shot LOSO validation, the mean $R^{2}$ remained below zero for all five feature configurations. Removing morphology therefore produced only a limited change in zero-shot performance relative to the all-feature configuration.

The morphology-excluded configuration was retained as the primary feature configuration in the subsequent subject-specific calibration analysis. The five feature profiles and their evaluation under both blocked temporal and zero-shot LOSO validation correspond to the current ablation protocol.

### 4.6. Subject-Specific Calibration Results

Subject-specific supervised calibration was evaluated using the initial 30 s of data from each held-out participant. The global ExtraTrees model and fold-specific feature-selection procedure were identical to those used in the zero-shot LOSO evaluation. A matched zero-shot baseline was calculated on the post-calibration interval, and all calibration methods were evaluated using the same target timestamps.

On the matched post-calibration interval, the zero-shot model achieved mean $R^{2}$ values of −0.233 ± 0.648 for $L_{\mathrm{Load}}$ and −0.141 ± 0.641 for $R_{\mathrm{Load}}$. The corresponding RMSE values were 0.360 and 0.341, respectively.

After regularized scale-and-offset calibration, the mean $R^{2}\;$values increased to 0.156 ± 0.234 for $L_{\mathrm{Load}}$ and 0.231 ± 0.287 for $R_{\mathrm{Load}}$. The corresponding RMSE values decreased to 0.306 and 0.287, respectively.

With the additional temporal adjustment, regularized temporal, scale, and offset calibration achieved mean $R^{2}$ values of 0.358 ± 0.232 for $L_{\mathrm{Load}}$ and 0.444 ± 0.245 for $R_{\mathrm{Load}}$. The corresponding RMSE values were 0.265 and 0.243, and the MAE values were 0.222 and 0.198, respectively. The complete comparison is presented in Table 6.

Compared with the matched zero-shot baseline, both calibration strategies increased the mean $R^{2}$ and reduced the mean RMSE and MAE for both plantar-load targets. The temporal, scale, and offset calibration achieved the highest mean $R^{2}$ among the three evaluated methods.

**Figure 4:**
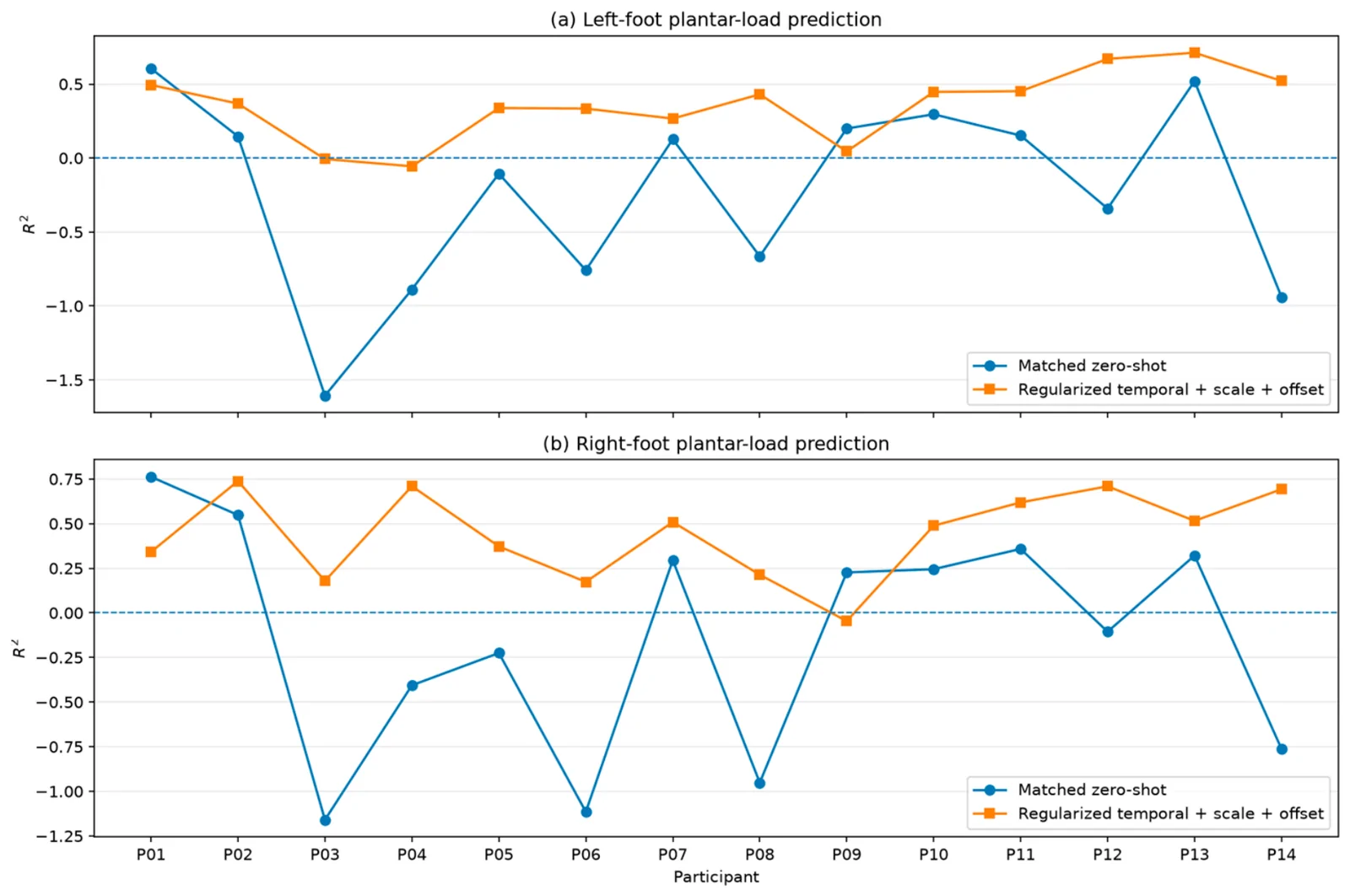
Individual-level coefficient of determination ($R^{2}$) before and after subject-specific calibration: (**a**) left-foot plantar-load prediction; (**b**) right-foot plantar-load prediction. Matched zero-shot predictions and predictions obtained after regularized temporal, scale, and offset calibration are shown for each held-out participant. The dashed horizontal line indicates $R^{2}=0$.

Figure 4 presents the individual-level $R^{2}$ values before and after regularized temporal, scale, and offset calibration. Compared with the matched zero-shot baseline, calibration improved $R^{2}$ for 12 of the 14 participants for each plantar-load target, although the magnitude of improvement varied across individuals.

## 5. Discussion

### 5.1. Prediction Performance and Cross-Subject Generalization

The present study investigated the estimation of bilateral plantar loads from biomechanical features derived from monocular video-estimated body pose during treadmill walking. Under blocked temporal five-fold cross-validation, ExtraTrees achieved the highest mean $R^{2}$ and the lowest RMSE and MAE among the four evaluated regression models. Using the morphology-excluded feature configuration, the mean $R^{2}$ values were 0.762 for $L_{\mathrm{Load}}$ and 0.772 for $R_{\mathrm{Load}}$. These results demonstrate that pose-derived biomechanical features contain information relevant to plantar-load estimation during temporally held-out walking observations from participants represented in the training data.

Previous studies have investigated the estimation of ground reaction forces from kinematic information using wearable sensors and markerless motion-capture systems [9,11]. Scott et al. further demonstrated the use of human pose for predicting plantar-pressure distributions [10]. Building on this research direction, the present study examined bilateral total plantar-load estimation using biomechanical features derived from a single video viewpoint and conventional machine-learning regressors. However, differences in prediction targets, movement tasks, and evaluation protocols prevent direct comparisons of the reported performance metrics.

In contrast to blocked temporal validation, zero-shot leave-one-subject-out (LOSO) validation yielded mean $R^{2}$ values of −0.252 and −0.180 for the left- and right-foot targets, respectively, with considerable inter-individual variation. This performance difference highlights the importance of distinguishing between temporally held-out observations from previously represented participants and observations from entirely unseen individuals. Previous methodological research has emphasized the importance of subject-independent evaluation when assessing the generalizability of machine-learning models in human movement biomechanics [29].

The observed results suggest that the relationship between pose-derived features and plantar loading may vary across individuals. Differences in walking patterns and individual loading characteristics may contribute to this variation, motivating the investigation of subject-specific calibration.

### 5.2. Contribution of Biomechanical Feature Groups

The feature-group ablation analysis demonstrated that posture-related features provided substantial predictive information under the present experimental conditions. Under blocked temporal validation, the posture-only configuration achieved mean $R^{2}$ values of 0.773 and 0.777 for the left- and right-foot targets, respectively, compared with 0.577 and 0.621 for the motion-only configuration. Because the posture-only configuration included temporal extensions, its performance reflects information from both body configuration and recent postural changes rather than isolated static poses.

Removing the three static morphology variables had only a small effect on blocked-validation performance, whereas the morphology-only configuration achieved considerably lower mean $R^{2}$ values of 0.097 and 0.094. Although anthropometric characteristics have been associated with plantar-pressure patterns during walking [30], the static morphology variables examined here provided limited information for predicting temporal variations in bilateral plantar loads when used alone.

Under zero-shot LOSO validation, all five feature configurations produced negative mean $R^{2}$ values. Excluding morphology therefore did not substantially improve cross-subject prediction, suggesting that participant-specific differences may extend beyond the three explicit static morphology variables. The morphology-excluded configuration was retained because it preserved similar blocked-validation performance while avoiding direct reliance on these measurements.

Overall, the results support the use of posture-related and short-term temporal information for plantar-load estimation. However, the observed feature contributions represent predictive associations rather than causal effects of individual biomechanical variables.

### 5.3. Subject-Specific Calibration and Practical Implications

Subject-specific supervised calibration was investigated to improve prediction for previously unseen participants without retraining the underlying ExtraTrees model. Previous research has shown that a limited amount of individual calibration data can improve subject-independent performance in wearable-sensor-based gait classification [31]. Motivated by this general principle, the present study investigated a lightweight calibration strategy for bilateral plantar-load regression. Calibration parameters were estimated using the initial 30 s of reference-load data from each held-out participant and subsequently applied to a separate post-calibration interval.

On the matched evaluation interval, the mean zero-shot $R^{2}$ values were −0.233 and −0.141 for the left- and right-foot targets, respectively. Regularized scale-and-offset calibration increased these values to 0.156 and 0.231. With additional temporal adjustment, the mean $R^{2}$ values increased further to 0.358 and 0.444, accompanied by reductions in RMSE and MAE. The temporal, scale, and offset calibration improved $R^{2}$ for 12 of the 14 participants for each target, although individual responses varied.

The improvement obtained through scale-and-offset calibration suggests that differences in prediction magnitude and baseline contribute to the discrepancy between global-model predictions and individual reference loads. The additional improvement obtained through temporal adjustment indicates that waveform alignment is also relevant to prediction performance. However, the estimated temporal shifts are empirical calibration parameters and should not be interpreted as direct measurements of synchronization error.

Conventional plantar-pressure measurement requires dedicated sensing devices [1,5,7]. In the proposed framework, a short period of reference-load measurement is used to adapt the global model to a new participant, after which calibrated predictions can be generated from video-derived features without continuous plantar-load measurements.

These findings support the potential of short supervised calibration for improving average plantar-load prediction under the present experimental conditions. However, further evaluation of calibration duration, parameter stability, and performance across different walking conditions is required before broader practical application.

### 5.4. Limitations

Several limitations should be considered. First, the present analysis included 14 healthy adults aged 22–34 years and 50,464 frame-level observations. Although the dataset provided substantial temporal information, the number of independent participants remained limited.

Second, the experiments were restricted to normal treadmill walking at 3 km/h under controlled conditions with a fixed camera viewpoint. Other walking speeds, overground walking, and more complex daily activities were not evaluated. Previous research has identified camera viewing angle and distance as factors affecting single-camera gait measurements [13]. Therefore, the performance observed under the present experimental configuration may not directly transfer to different walking conditions or recording environments.

Third, the biomechanical features were derived from body landmarks estimated using a single monocular camera. Landmark estimation errors, partial occlusion, and limited depth information may affect prediction accuracy. Furthermore, the framework estimates bilateral total plantar loads rather than regional plantar-pressure distributions.

Finally, subject-specific calibration required an initial 30 s period of reference-load measurement. Moreover, the temporal-adjustment procedure permitted shifts of up to ±300 ms, potentially requiring video information acquired after the target prediction time. The temporal-calibration results therefore represent offline evaluation rather than verified real-time prediction performance.

## 6. Conclusions

This study developed a low-cost, monocular video-based framework for estimating bilateral plantar loads from pose-derived biomechanical features during treadmill walking. Using data from 14 healthy adult participants, the framework combined posture-related, motion-related, and short-term temporal features with machine-learning regression. Among the four evaluated models, ExtraTrees achieved the highest mean predictive performance under blocked temporal five-fold cross-validation, with mean $R^{2}$ values of 0.762 and 0.772 for the left- and right-foot plantar loads, respectively.

Feature-group ablation showed that the posture-only and morphology-excluded configurations achieved performance similar to that of the all-feature configuration under blocked temporal validation. Excluding the three static morphology variables had only a small effect on prediction performance. Under zero-shot leave-one-subject-out (LOSO) validation, however, the mean $R^{2}$ values were −0.252 and −0.180 for the left- and right-foot targets, respectively, indicating limited generalization to previously unseen participants without individual calibration.

Using the initial 30 s of reference-load data for subject-specific supervised calibration improved the mean prediction performance on the matched post-calibration interval. Regularized temporal, scale, and offset calibration increased the mean $R^{2}$ values from −0.233 to 0.358 for the left-foot target and from −0.141 to 0.444 for the right-foot target, while reducing the corresponding prediction errors. These findings demonstrate that short supervised calibration can improve the average plantar-load prediction performance for previously unseen participants without retraining the underlying regression model.

Overall, the proposed framework demonstrates the potential of monocular video-derived biomechanical features for bilateral plantar-load estimation during controlled treadmill walking. After initial subject-specific calibration, predictions can be generated from video-derived information without continuous wearable-sensor measurements. Future work will evaluate the framework in larger and more diverse participant cohorts, across additional walking speeds, overground walking conditions, and different camera viewpoints and recording environments. Further investigations will examine shorter calibration intervals, the stability of calibration parameters across recording sessions, and strictly causal temporal adjustment for real-time implementation. As larger and more diverse datasets become available, the framework may also be extended toward estimating detailed regional plantar-pressure distributions.

## Figures and Tables

**Table 1:** Core pose-derived biomechanical variables and their definitions.

Category	Variable(s)	Biomechanical Meaning	Calculation/Definition
Posture	L/R_knee_angle	Bilateral knee configuration	Angle between the hip-to-knee and ankle-to-knee vectors on the corresponding side, calculated using the hip, knee, and ankle landmarks.
Posture	L/R_hip_angle	Bilateral hip configuration	Angle between the shoulder-to-hip and knee-to-hip vectors on the corresponding side, calculated using the shoulder, hip, and knee landmarks.
Posture	trunk_tilt_angle	Trunk orientation	Angle between the shoulder-center–hip-center vector and the vertical axis.
Posture	L/R_ankle_height_rel	Relative vertical ankle position	Vertical coordinate of the corresponding ankle landmark relative to the hip center, after body-height normalization.
Posture	ankle_distance	Bilateral ankle separation	Euclidean distance between the left and right ankle landmarks in the normalized coordinate system.
Motion	v_pelvis	Overall pelvis translational motion	Magnitude of the temporal rate of change in the normalized hip-center position.
Motion	pelvis_vy	Vertical pelvis motion	Vertical component of the temporal rate of change in the normalized hip-center position.
Motion	pelvis_ay	Vertical pelvis acceleration	Temporal rate of change in pelvis_vy.
Motion	v_L/R_ankle	Bilateral ankle translational motion	Magnitude of the temporal rate of change in the normalized position of the corresponding ankle landmark.
Motion	L/R_ankle_vy	Vertical ankle motion	Vertical component of the temporal rate of change in the normalized position of the corresponding ankle landmark.
Motion	L/R_ankle_ay	Vertical ankle acceleration	Temporal rate of change in the corresponding ankle vertical velocity.
Motion	v_L/R_knee	Bilateral knee translational motion	Magnitude of the temporal rate of change in the normalized position of the corresponding knee landmark.
Motion	L/R_knee_vy	Vertical knee motion	Vertical component of the temporal rate of change in the normalized position of the corresponding knee landmark.
Motion	L/R_knee_ay	Vertical knee acceleration	Temporal rate of change in the corresponding knee vertical velocity.
Motion	L/R_knee_angle_vel	Bilateral knee angular motion	Temporal rate of change in the corresponding knee angle.
Motion	L/R_hip_angle_vel	Bilateral hip angular motion	Temporal rate of change in the corresponding hip angle.
Morphology	trunk_length	Relative trunk dimension	Euclidean distance between the shoulder center and the hip center in the normalized coordinate system.
Morphology	hip_width	Relative pelvic width	Euclidean distance between the left and right hip landmarks in the normalized coordinate system.
Morphology	shoulder_width	Relative shoulder width	Euclidean distance between the left and right shoulder landmarks in the normalized coordinate system.

Note: L/R denotes the corresponding left- and right-side variables. The shoulder center and hip center are defined as the midpoints of the corresponding bilateral landmarks. Position- and distance-related variables are derived from the preprocessed, hip-centered, and body-height-normalized pose coordinates. Temporal rates of change are calculated within each participant–trial sequence. The three morphology-related variables are retained for the feature-group ablation analysis but excluded from the primary prediction configuration.

**Table 2:** ExtraTrees Model Hyperparameters:

Hyperparameter	Value
n_estimators	150
max_features	0.75
min_samples_leaf	2
random_state	42

**Table 3:** Performance comparison of the four regression models under blocked temporal five-fold cross-validation.

Model	Target	*R* ^2^	RMSE	MAE
CatBoost	$L_{\mathrm{Load}}$	0.634 ± 0.063	0.206 ± 0.017	0.163 ± 0.012
CatBoost	$R_{\mathrm{Load}}$	0.647 ± 0.075	0.199 ± 0.020	0.156 ± 0.012
XGBoost	$L_{\mathrm{Load}}$	0.708 ± 0.073	0.183 ± 0.022	0.132 ± 0.015
XGBoost	$R_{\mathrm{Load}}$	0.712 ± 0.082	0.179 ± 0.024	0.129 ± 0.016
LightGBM	$L_{\mathrm{Load}}$	0.713 ± 0.074	0.182 ± 0.023	0.130 ± 0.015
LightGBM	$R_{\mathrm{Load}}$	0.717 ± 0.082	0.178 ± 0.025	0.126 ± 0.016
**ExtraTrees**	$L_{\mathrm{Load}}$	**0.762 ± 0.068**	**0.165 ± 0.023**	**0.117 ± 0.016**
**ExtraTrees**	$R_{\mathrm{Load}}$	**0.772 ± 0.073**	**0.159 ± 0.024**	**0.110 ± 0.015**

Values are presented as mean ± standard deviation across the five validation folds. RMSE and MAE are expressed in lbf/kg. Bold values indicate the best performance among the four regression models for each target.

**Table 4:** Zero-shot LOSO prediction performance across the 14 held-out participants.

Target	*R* ^2^	RMSE	MAE
$L_{\mathrm{Load}}$	−0.252 ± 0.592	0.364 ± 0.069	0.331 ± 0.067
$R_{\mathrm{Load}}$	−0.180 ± 0.573	0.350 ± 0.073	0.312 ± 0.068

Note: Values are presented as mean ± standard deviation across the 14 held-out participants. RMSE and MAE are expressed in lbf/kg.

**Table 5:** Feature-group ablation results under blocked temporal and zero-shot LOSO validation.

Feature Configuration	Blocked L *R*^2^	Blocked R *R*^2^	LOSO L *R*^2^	LOSO R *R*^2^
All features	0.768 ± 0.065	0.777 ± 0.070	−0.269 ± 0.583	−0.172 ± 0.555
Without morphology	0.762 ± 0.068	0.772 ± 0.073	−0.252 ± 0.592	−0.180 ± 0.573
Posture only	0.773 ± 0.068	0.777 ± 0.074	−0.252 ± 0.582	−0.178 ± 0.545
Motion only	0.577 ± 0.056	0.621 ± 0.069	−0.254 ± 0.545	−0.126 ± 0.543
Morphology only	0.097 ± 0.036	0.094 ± 0.041	−0.277 ± 0.306	−0.254 ± 0.229

**Table 6:** Zero-shot and subject-specific calibrated prediction performance on the matched post-calibration interval.

Method	L $R^{2}$	L RMSE	L MAE	R $R^{2}$	R RMSE	R MAE
Zero-shot	−0.233 ± 0.648	0.360 ± 0.084	0.327 ± 0.080	−0.141 ± 0.641	0.341 ± 0.091	0.306 ± 0.083
Regularized scale + offset	0.156 ± 0.234	0.306 ± 0.048	0.282 ± 0.053	0.231 ± 0.287	0.287 ± 0.060	0.261 ± 0.065
Regularized temporal + scale + offset	0.358 ± 0.232	0.265 ± 0.043	0.222 ± 0.045	0.444 ± 0.245	0.243 ± 0.050	0.198 ± 0.047

## Data Availability

The data presented in this study are available on reasonable request from the corresponding author due to privacy and ethical restrictions. The raw dataset includes video recordings of human participants and cannot be made publicly available because of participant privacy and confidentiality considerations.
